# Treatment Outcomes With an Oral Short Course Regimen for Rifampicin-resistant Tuberculosis in a High HIV Prevalence, Programmatic Setting in South Africa

**DOI:** 10.1093/cid/ciaf112

**Published:** 2025-05-09

**Authors:** Jacob A M Stadler, Johanna Kuhlin, Síle F Molloy, Nomfuneko Mtwa, Cindy Hayes, David Stead, Gary Maartens, Robin Warren, Graeme Meintjes, Sean Wasserman

**Affiliations:** Wellcome Discovery Research Platforms in Infection, Centre for Infectious Diseases Research in Africa, Institute of Infectious Disease and Molecular Medicine, University of Cape Town, Cape Town, South Africa; Department of Medicine, University of Cape Town, Cape Town, South Africa; Wellcome Discovery Research Platforms in Infection, Centre for Infectious Diseases Research in Africa, Institute of Infectious Disease and Molecular Medicine, University of Cape Town, Cape Town, South Africa; Department of Medicine Solna, Karolinska Institutet, Stockholm, Sweden; Department of Infectious Diseases, Karolinska University Hospital, Stockholm, Sweden; Institute for Infection and Immunity, City St George's, University of London, London, United Kingdom; Department of Health, Eastern Cape Province, South Africa; National Health Laboratory Service, Port Elizabeth, South Africa; Wellcome Discovery Research Platforms in Infection, Centre for Infectious Diseases Research in Africa, Institute of Infectious Disease and Molecular Medicine, University of Cape Town, Cape Town, South Africa; Department of Medicine, University of Cape Town, Cape Town, South Africa; Wellcome Discovery Research Platforms in Infection, Centre for Infectious Diseases Research in Africa, Institute of Infectious Disease and Molecular Medicine, University of Cape Town, Cape Town, South Africa; Division of Clinical Pharmacology, Department of Medicine, University of Cape Town, Cape Town, South Africa; DSI-NRF Centre of Excellence for Biomedical Tuberculosis Research/South African Medical Research Council Centre for Tuberculosis Research, Division of Molecular Biology and Human Genetics, Faculty of Medicine and Health Sciences, Stellenbosch University, Cape Town, South Africa; Wellcome Discovery Research Platforms in Infection, Centre for Infectious Diseases Research in Africa, Institute of Infectious Disease and Molecular Medicine, University of Cape Town, Cape Town, South Africa; Department of Medicine, University of Cape Town, Cape Town, South Africa; Blizard Institute, Queen Mary University of London, London, United Kingdom; Wellcome Discovery Research Platforms in Infection, Centre for Infectious Diseases Research in Africa, Institute of Infectious Disease and Molecular Medicine, University of Cape Town, Cape Town, South Africa; Institute for Infection and Immunity, City St George's, University of London, London, United Kingdom; Division of Infectious Diseases and HIV Medicine, Department of Medicine, University of Cape Town, Cape Town, South Africa

**Keywords:** drug-resistant tuberculosis, rifampicin-resistant tuberculosis, short oral regimen, outcomes, HIV

## Abstract

**Background:**

Bedaquiline-based oral short-course regimens (SCR) for rifampicin-resistant tuberculosis (RR-TB) are highly effective in clinical trials but outcomes in programmatic settings may be more modest. We evaluated clinical and bacteriological outcomes with a seven-drug, linezolid-containing SCR in a high-burden programmatic setting.

**Methods:**

This prospective cohort study enrolled adults with newly diagnosed RR-TB who were started on the oral SCR in the Eastern Cape Province, South Africa. The primary outcome was World Health Organization-defined end-of-treatment success. Secondary outcomes were TB-free survival (composite of alive, absence of a positive *Mycobacterium tuberculosis* culture, and treatment completed or in care) at 18 months and time to sputum culture conversion (SCC).

**Results:**

In total, 248 participants were included, 173 (69.8%) of whom were human immunodeficiency virus (HIV) positive. Culture conversion by 90 days was 96.8% (median time to SCC: 29 days, 95% confidence interval [CI]: 27–31). Treatment success was 37.5% (93/248). Reasons for unsuccessful treatment included switching to individualised regimens (35.1%, 87/248), loss to follow-up (19.4%, 48/348), and death (8.1%, 20/248). At 18 months, 157 (63.3%) participants achieved TB-free survival, with a cumulative mortality of 21.6% (95% CI: 16.1–29.0). Baseline 3+ smear (adjusted odds ratio [aOR]: 3.38, 95% CI: 1.28–8.95), higher age (aOR: 1.05, 1.01–1.08), and lower albumin (aOR: 0.94, 0.88–0.99), but not HIV status, were associated with unfavourable outcome at 18 months.

**Conclusions:**

The oral SCR performed poorly in a high-burden TB programme. Strategies to support the implementation of effective new regimens for RR-TB are needed to translate outcomes from clinical trials into practice.

## Teaser

A standardized 9-month oral regimen for rifampicin-resistant tuberculosis achieved low treatment success in a programmatic setting due to a high proportion changing to individualized regimens, loss to follow-up, and death. Outcomes were similar irrespective of HIV status.

The World Health Organization (WHO) recommends several shorter oral bedaquiline-containing regimens for the treatment of rifampicin-resistant tuberculosis (RR-TB), including the 6-month bedaquiline-pretomanid-linezolid-moxifloxacin (BPaL/M) regimen and three 9-month regimens from the endTB trial [1, 2]. The BPaL/M regimen is preferred as it has a shorter duration (6 months) and a lower pill burden and can be used irrespective of fluoroquinolone resistance. Alternative 7-drug regimens of 9–11 months duration can be used in the absence of fluoroquinolone resistance, extensive lung involvement, or complicated extra-pulmonary disease. One of these 9-month regimens was introduced into the South African National Tuberculosis Program (SA-NTP) in 2018, pioneering the use of shortened fully oral treatment for RR-TB [3]. The regimen is a modified version of the STREAM I trial regimen [4] (which provided the first definitive evidence for the effectiveness of shorter RR-TB treatment) substituting bedaquiline for an injectable agent and linezolid (provided for 2 months) for ethionamide, thus including all 3 WHO group A drugs.

At the time of implementation, there was no direct evidence for the effectiveness of this regimen in TB programmes. Its inclusion in WHO guidelines in 2022 was primarily based on data from the SA-NTP, which reported 64% treatment success in a programmatic setting [1, 5]. However, such registry data lacks detailed clinical information, longitudinal treatment characterisation, and precise determination of treatment outcomes, limiting understanding of actual regimen performance.

We aimed to evaluate the effectiveness of the 7-drug oral short course regimen (SCR) in a programmatic setting with high human immunodeficiency virus (HIV) prevalence. Here we describe clinical and bacteriological outcomes through 18 months after treatment initiation and explore predictors of unfavourable outcome and time to sputum culture conversion, including the impact of HIV.

## METHODS

### Design, Setting, and Participants

We conducted a prospective observational study at a centralised referral facility for drug-resistant TB in the Eastern Cape Province, South Africa. The facility serves a mixed urban and rural population and provides both in- and outpatient care. Consenting patients older than 15 years with newly diagnosed (≤30 days on treatment) pulmonary RR-TB who were started on the oral SCR in routine care were consecutively enrolled. Study personnel played no role in treatment decisions. Recruitment commenced in January 2021, and follow-up visits concluded in August 2023.

### Data and Procedures

Participants attended 7 study visits through the end of treatment or 12 months after initiation of treatment, whichever occurred first. Sputum for *Mycobacterium tuberculosis* (Mtb) microscopy and culture was collected bi-weekly until week 8 and monthly thereafter. Liquid sputum cultures were performed at the provincial reference TB laboratory using the mycobacterial growth indicator tube system (Bactec MGIT 960, BD Biosciences, USA). All additional Mtb smear and culture results originating from routine care were also recorded. All drug susceptibility testing (DST) results from routine care were collected. This included the initial detection of rifampicin resistance (primarily using GeneXpert MTB/Rif), followed by genotypic testing for isoniazid, fluoroquinolones and aminoglycosides (Hain Line Probe Assay). Confirmatory phenotypic DST for isoniazid was done if the genotype was susceptible. Bedaquiline and linezolid resistance testing was done only if fluoroquinolone resistance was detected, or if clinically indicated. Month 18 outcomes were verified through a combination of in-person or telephonic contact with participants or next of kin, a comprehensive medical record review (hospital folders, electronic pharmacy records and National Health Laboratory Services database) as well as a National Death Registry search.

### Outcome Definitions

The primary outcome was end-of-treatment success based on the WHO Reporting Framework 2021, providing a measure of regimen performance [6]. Successful treatment was defined as the sum of treatment completion (receipt of at least 8 months and no more than 13 months of the oral SCR) and cure (sustained culture conversion and absence of unsuccessful treatment). Unsuccessful treatment included death, loss to follow up (treatment interruption ≥2 consecutive months), or regimen failure (permanent change or discontinuation of ≥2 drugs of the standardised oral SCR).

We continued to follow participants who switched to an individualised regimen and those lost to follow-up with subsequent return to care. To provide a patient-centred measure of clinical effectiveness, irrespective of treatment completion status or regimen, we developed a secondary outcome measure termed “TB-free survival”, a composite of being alive, the absence of a positive sputum culture at last assessment, and treatment completed or in care through 18 months from study entry (Supplementary Table 1).

Culture conversion was defined as 2 consecutive negative cultures taken at least 7 days apart, after a positive culture at baseline, and after receiving a minimum of 7 days of RR-TB treatment. A positive culture at baseline was defined as a positive Mtb culture from 60 days before to 30 days after treatment initiation. Inability to produce sputum was considered equivalent to a negative culture.

### Sample Size Considerations

Initial sample size calculation was based on comparing efficacy of the oral SCR to historical controls treated with the short, injectable-containing regimen recommended by the WHO at the time. Due to changes in clinical practice, this comparison was no longer relevant. A post hoc power calculation was performed based on outcomes from SA-NTP registry data [5], demonstrating that our sample size was able to detect treatment success of up to 60% with the SCR with 6% precision and 95% confidence.

### Statistical Analysis

The primary outcome was the proportion of participants with WHO-defined treatment success at the end of treatment with the oral SCR, with pre-specified comparison by HIV status, analysed for the full population, that is, all who started the oral SCR (intention-to-treat principle). A secondary, modified intention-to-treat (mITT) analysis was performed by excluding individuals considered ineligible for the regimen due to preexisting contraindications (according to local guidelines) not known at the time of treatment initiation. TB-free survival at 18 months after treatment initiation was analyzed for the full population. The proportion with treatment success and TB-free survival was computed and compared by HIV status using the *z*-test for 2 independent proportions.

Time-to-event outcomes, including culture conversion, loss to follow-up, and mortality were estimated using the Kaplan–Meier survival function, and groups were compared using restricted mean survival time and the log-rank test. Cox proportional hazards regression was used to explore factors associated with time to sputum culture conversion. HIV status was fixed as the main covariate of interest in the model. Other candidate variables for the full model were selected based on theoretical grounds and significant univariable associations (*P* < .2). A final model was derived using augmented backward elimination [7], with a 0.05 significance level for removal and 10% change-in-estimate criterion for retention in the model. The population for this model therefore included all participants with a positive culture at baseline (n = 203). Missing data were imputed using multiple imputation with chained equations.

Predictors of unfavourable outcome (failure to achieve TB-free survival at 18 months) were explored using binary logistic regression. Individuals with unassessable outcome status were excluded. Variable selection for the full model was primarily theory-driven, with HIV (categorised by viral suppression) to be retained in the model and clinically relevant markers of disease severity/progression or comorbidity as potential confounders. Additionally, statistically significant (*P* ≤ .05) univariable associations were included. Missing data and model selection were handled as described for Cox regression. Analysis was performed using Stata, version 17.

### Ethics

The study protocol was approved by the University of Cape Town Human Research Ethics Committee (REF. 690/2019) and the Eastern Cape Department of Health. Written informed consent was obtained before study participation.

## RESULTS

### Study Population and Characteristics

**Figure 1.**
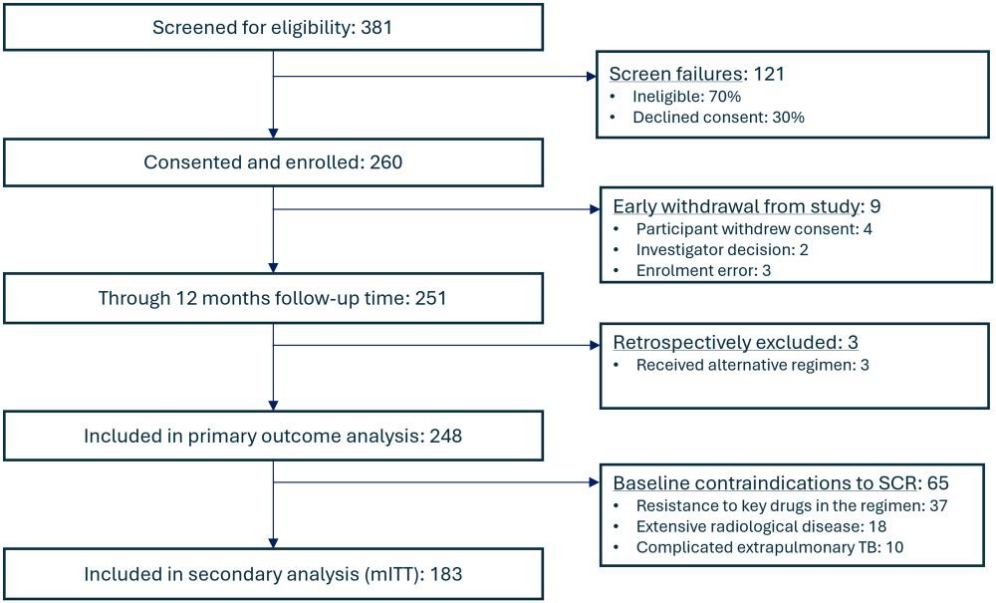
CONSORT diagram of analysis populations.

We enrolled 260 participants between January 2021 and July 2022, 12 of whom were excluded from analysis due to early study discontinuation/withdrawal or receiving a non-standard oral SCR, leaving 248. A further 65 (26.2%) were excluded for the mITT analysis (Figure 1). Although all participants started treatment with the standardized oral SCR, linezolid was initially omitted in 31/248 (12.5%) due to preexisting contraindications (mainly severe anaemia). In addition, 98 participants received a secondary, individualized regimen at some point during follow-up (Supplementary Table 2). All participants had rifampicin-resistant disease at baseline, with additional resistance to isoniazid confirmed in 126/248 (50.8%); 22/248 (8.9%) were also resistant to fluoroquinolones (Supplementary Figure 1). Prevalence of HIV coinfection was 69.8% (173/248) with a median CD4 cell count of 102 cells/mm^3^ (interquartile range [IQR]: 33.5–240) and 23.7% (41/173) virological suppression (Table 1). Those not already on antiretroviral treatment (ART) at the time of TB treatment initiation were all subsequently initiated on ART (Supplementary Table 3; median time to ART initiation: 15.5 days, min-max: 7–61 days).

**Table 1.** Baseline Characteristics of the Study Population

Values Given as n (%)	All n = 248 (100)	HIV-negative n = 75 (30.2)	HIV-positive n = 173 (69.8)
Sex			
Male	146 (58.9)	57 (75.0)	89 (51.7)
Age, years (median, IQR)	38 (30.5–45)	39 (30–49)	38 (31–44)
Body mass index (mean, SD)	19.6 (4.9)	19.4 (4.7)	19.7 (5.0)
TB history			
No previous TB	131 (52.8)	49 (65.3)	82 (47.4)
Previous DS-TB	117 (47.2)	26 (34.7)	91 (52.6)
ART	…	…	
On ART	…	…	107 (61.9)
Not on ART (ART naive)	…	…	17 (9.8)
Not on ART (ART experienced)	…	…	49 (28.3)
Timing of ART (re)initiation, days from TB treatment initiation (median, min–max)	…	…	15.5 (7–61)
CD4 cell count, cells/mm^3^ (median, IQR)	…	…	102 (33.5–240)
≤200	…	…	114 (65.9)
>200	…	…	50 (28.9)
No data	…	…	9 (5.2)
HIV viral load (window: ±6 m)	…	…	
Suppressed (<50 copies/mL)	…	…	41 (23.7)
Low-level viraemia (50–1000 copies/mL)	…	…	26 (15.0)
Unsuppressed (>1000 copies/mL)	…	…	79 (45.7)
No data	…	…	27 (15.6)
Initial treatment setting			
Outpatient	103 (41.5)	41 (54.6)	62 (35.8)
Inpatient (hospitalised)	145 (58.5)	34 (45.3)	111 (64.2)
Functional status (ECOG score)			
Grade 0–2	215 (86.7)	71 (94.7)	144 (83.2)
Grade 3–4	33 (13.3)	4 (5.3)	29 (13.3)
Fluoroquinolone resistance			
Resistant	22 (8.9)	7 (9.3)	15 (8.7)
Sputum culture status			
Positive	199 (80.2)	64 (85.3)	135 (78.0)
Sputum smear grade			
Negative	108 (43.6)	24 (32.0)	84 (48.6)
+	41 (16.5)	15 (20.0)	26 (15.0)
++	42 (16.9)	17 (22.7)	25 (14.5)
+++	54 (21.8)	19 (25.3)	35 (20.2)
No data	3 (1.2)	0 (0.0)	3 (1.7)
GeneXpert Mtb/Rif cycle threshold (median, IQR)	16.4 (16.2–18.6)	16.3 (16.2–18.3)	16.4 (16.2–19.0)
≤18	145 (58.5)	47 (62.7)	98 (56.7)
>18	60 (24.2)	16 (21.3)	44 (25.4)
No data	43 (17.3)	12 (16.0)	31 (17.9)
Site of disease			
Pulmonary TB	241 (97.2)	75 (100.0)	166 (96.0)
Pulmonary and extra-pulmonary TB	7 (2.8)	0 (0.0)	7 (4.1)
Linezolid treatment duration, days (median, IQR)	69 (57–113)	70 (61–148)	67 (55–98)
Diabetes (HbA1c >6.5%)			
No	175 (70.6)	46 (61.3)	129 (74.6)
Yes	28 (11.3)	13 (17.3)	15 (8.7)
No data	45 (18.2)	16 (21.3)	29 (16.8)
Hemoglobin, g/dL (median, IQR)	10.5 (8.7–12.2)	11.8 (9.6–13.2)	9.9 (8.4–11.8)
<8	30 (12.1)	3 (4.0)	27 (15.6)
Albumin, g/L (median, IQR)	29 (24–35)	34 (27–37)	28 (23–33)
Highest level of education			
Primary school only or no formal education	59 (23.8)	27 (36.0)	32 (18.5)
High school without matriculating	134 (54.0)	34 (45.3)	100 (57.8)
Matric or higher	55 (22.2)	14 (18.7)	41 (23.7)
Employment status			
Working (including self-employed)	76 (30.7)	21 (28.0)	55 (31.8)
Unemployed (looking for work)	99 (39.9)	28 (37.3)	71 (41.0)
Scholar/student	11 (4.4)	8 (10.7)	3 (1.7)
Other	62 (25.0)	18 (24.0)	44 (25.0)
Social grant recipient			
No	161 (64.9)	50 (66.7)	111 (64.2)
Yes	87 (35.1)	25 (33.3)	62 (35.8)
Household income (monthly)			
<R1000	52 (21.0)	14 (18.7)	38 (22.0)
R1000–R5000	138 (55.7)	47 (62.7)	91 (52.6)
R5000–R10000	30 (12.1)	8 (10.7)	22 (12.7)
>R10000	28 (11.3)	6 (8.0)	22 (12.7)
CAGE score (alcohol use disorder screening)			
0–2 (Low risk)	169 (68.2)	59 (78.7)	110 (63.6)
3–4 (High risk)	79 (31.9)	16 (21.3)	63 (36.4)

Abbreviations: ART, antiretroviral therapy; CAGE, acronym for “Cutting down, Annoyance by criticism, Guilty feeling, and Eye-openers”; DS-TB, drug-susceptible tuberculosis; ECOG, Eastern Cooperative Oncology Group; HbA1c, hemoglobin A1c; IQR, interquartile range; SD, standard deviation.

### Regimen Outcomes

WHO-defined treatment success was achieved in 93/248 (37.5%) (Table 2). Reasons for unsuccessful treatment included switching to an individualized regimen (87/248, 35.1%), loss to follow-up (48/248, 19.4%) and death during treatment (20/248, 8.1%). Regimen changes were mainly due to preexisting contraindications to treatment with an oral SCR as per national guidelines: 37/248 (14.9%) with baseline resistance to key drugs, 18/248 (7.3%) with extensive lung disease and 10/248 (4.0%) with extra-pulmonary disease. Poor clinical or bacteriological response (6/248, 2.4%) and adverse drug reactions (8/248, 3.2%) were infrequent reasons for regimen failure. Only one instance (1/248, 0.4%) of treatment-emergent resistance was identified through routine DST: an acquired *katG* mutation in a patient with a baseline *inhA* mutation. There was no difference by HIV status in the proportion with a composite unsuccessful treatment outcome (111/173, 64.2% for HIV-positive vs 44/75, 58.7% for HIV-negative, *P* = .42) or death (15/173, 8.7% for HIV-positive vs 5/75, 6.6% for HIV-negative, *P* = .59). In the mITT population, the proportion treatment success was 49.2% (90/183), with no difference between HIV-positive (62/129, 48.1%) and HIV-negative participants (31/54, 57.4%, *P* = .25; Supplementary Table 4).

**Table 2.** Treatment Outcomes^a^ for the Oral SCR at the End of Treatment With the Regimen, Among All Participants Who Started the Regimen (n = 248)

Values Given as n (%)	All n = 248 (100)	HIV-negative n = 75 (30.2)	HIV-positive n = 173 (69.8)	*P* Value
Regimen successful (cured and treatment completed)	93 (37.5)	31 (41.3)	62 (35.8)	*P* = .42
Regimen unsuccessful	155 (62.5)	44 (58.7)	111 (64.2)	*P* = .42
Died	20 (8.1)	5 (6.7)	15 (8.7)	*P* = .59
Lost to follow-up^b^	48 (19.4)	13 (17.3)	35 (20.2)	*P* = .60
Treatment failed^c^ (permanent regimen change)	87 (35.1)	26 (34.7)	61 (35.3)	*P* = .93
Poor clinical and/or bacteriological response	6 (2.4)	1 (3.7)	5 (8.3)	
Adverse drug reaction	8 (3.2)	2 (7.4)	6 (10.0)	
Acquired resistance to drugs in the regimen	1 (0.4)	0 (0.0)	1 (1.7)	
Baseline resistance to drugs in the regimen	37 (14.9)	12 (15.8)	25 (14.5)	
Extrapulmonary TB	10 (4.0)	1 (3.7)	9 (5.2)	
Extensive lung disease	18 (7.3)	9 (11.8)	9 (5.2)	
Other	7 (2.8)	2 (2.6)	5 (2.9)	

Abbreviations: HIV, human immunodeficiency virus; TB, tuberculosis: WHO, World Health Organization.

^a^2021 WHO Reporting Framework.

^b^Lost to follow-up = Treatment interruption for ≥2 consecutive months.

^c^Treatment failed = A patient whose regimen was terminated or permanently changed. Regimen change was defined as permanent discontinuation or change of ≥2 drugs of the standardised short course regimen. Substitution with an equivalent drug (eg moxifloxacin for levofloxacin) was not considered a drug change.

### Month 18 Outcomes

**Figure 2.**
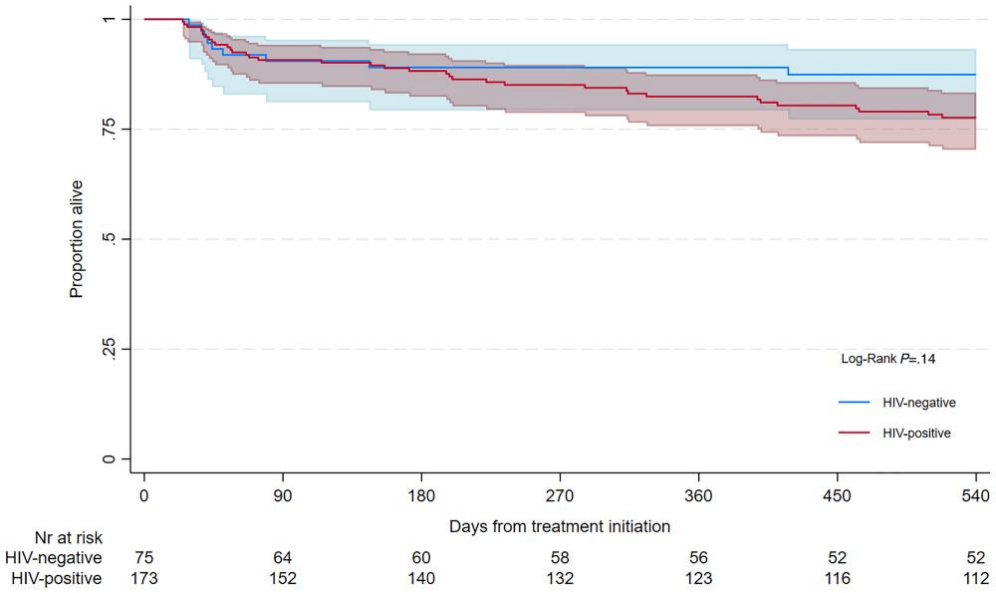
Survival through 18 m by HIV status, censored for loss to follow-up without return to care. Abbreviations: CI, confidence interval; HIV, human immunodeficiency virus.

TB-free survival (favourable outcome) at 18 months was observed in 157/248 (63.3%) participants and did not differ by HIV status (109/173, 63.0% for HIV-positive, *P* = .88) (Table 3). Of the 93 participants who successfully completed the oral SCR, 92 remained alive and without evidence of active TB (WHO-defined “sustained treatment success”) by month 18, while 1 had died. A total of 56/248 (22.6%) participants were still receiving treatment, 4/248 (1.6%) had evidence of active TB, and 45/248 (18.1%) had died by month 18 (cumulative incidence 21.6%, 95% CI: 16.1–29.0). More deaths occured among HIV-positive participants, though the difference was not statistically significant (36/173, 20.8% in HIV-positive vs 9/75, 12.0% in HIV-negative, *P* = .10). Survival time through 18 months also did not differ significantly by HIV status (log-rank *P* = .14; Figure 2).

**Table 3.** TB-free Survival at 18-months After Treatment Initiation, Irrespective of Regimen Used or Treatment Completion, Among All Participants Who Started the Short Regimen (n = 248)

Values Given as n (%)	All 248 (100)	HIV-negative 75 (30.2)	HIV-positive 173 (69.8)	*P* Value
Achieved	157 (63.3)	48 (64.0)	109 (63.0)	*P* = .88
Alive, TB-free and treatment complete	105 (42.3)	35 (46.7)	70 (40.5)	
Alive, TB-free and in care (treatment ongoing)	52 (21.0)	13 (17.3)	39 (22.5)	
NOT achieved	49 (19.8)	11 (14.7)	38 (22.0)	*P* = .18
Died	45 (18.1)	9 (12.0)	36 (20.8)	*P* = .10
NOT TB-free, but alive and in care (treatment ongoing)	4 (1.6)	2 (2.7)	2 (1.2)	
Unassessable (treatment NOT complete and NOT in care, ie, lost to follow-up)	42 (16.9)	16 (21.3)	26 (15.0)	*P* = .22

### Outcomes Following Treatment Interruption

**Figure 3.**
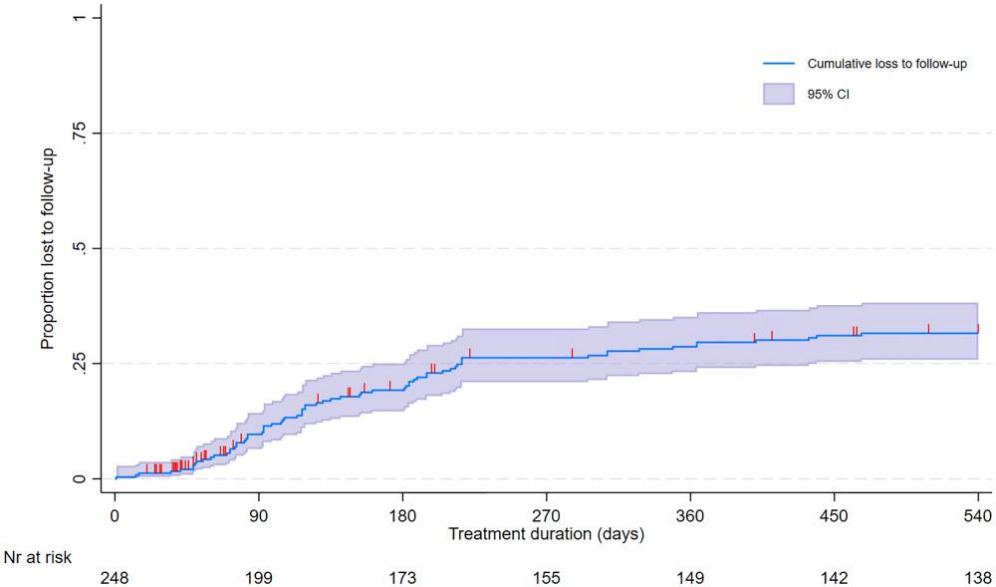
Cumulative loss to follow-up through 18 m, irrespective of the regimen used, censored for death or transfer out. Abbreviation: HIV, human immunodeficiency virus.

Patient-initiated treatment interruption for ≥2 consecutive months (WHO-defined “loss to follow-up”) occurred in 73 (29.4%) individuals (Figure 3), of whom 44/73 (60.3%) had returned to care within 18 months. The median treatment duration before interruption was 158 days (IQR: 103–238) and the median duration of interruption was 163 days (IQR: 90–218). Upon return to care, 10/44 (22.7%) participants were culture-positive and 16/44 (36.4%) did not reinitiate treatment because of sustained culture negativity. Based on DST data from routine care, 2 cases of acquired resistance were identified, one of which acquired bedaquiline resistance.

### Bacteriological Response

**Figure 4.**
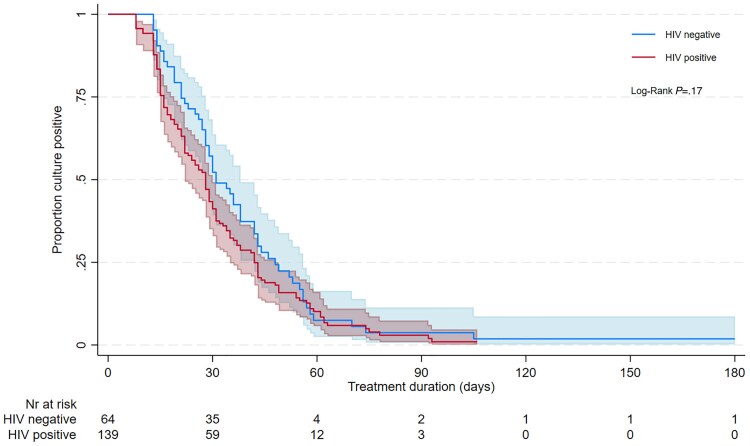
Time to sputum culture conversion through 6 m by HIV status. Abbreviation: HIV, human immunodeficiency virus.

In sum, 203/248 (81.8%) participants were sputum Mtb culture positive at baseline. The median time to sputum culture conversion was 29 days (95% CI: 27–31) and by 90 days the cumulative proportion with culture conversion was 96.8% (95% CI: 93.2–98.8). There was no difference in time to culture conversion between HIV-positive and -negative participants (log-rank *P* = .17; restricted mean survival time difference = −4.9 days, 95% CI: −11.3 to 1.5, *P* = .14; Figure 4), including after adjustment for HIV virological control at baseline (Supplementary Table 5). In the final multivariable model, high-grade (3+) sputum smear positivity at baseline was associated with a slower rate of sputum culture conversion (adjusted hazard ratio [aHR] = 0.64, 95% CI: .42–.95) and an initial period of in-hospital care was associated with faster culture conversion (aHR = 1.53, 95% CI: 1.09–2.15).

### Predictors of Unfavorable Outcome

By 18 months after treatment initiation, 49/248 (19.8%) participants had experienced an unfavourable study outcome (TB-free survival not achieved) and 42/248 (16.9%) were classified as unassessable and excluded (Table 3). After adjustment, HIV-positivity was not associated with an unfavourable outcome status at 18 months, irrespective of the level of viral suppression at baseline (Supplementary Table 3). Increasing age (adjusted odds ratio, aOR = 1.05, 95% CI: 1.01–1.08 per year), lower albumin (aOR = 0.94, 95% CI: .88–.99 per unit) and 3+ sputum smear positivity (aOR = 3.38, 95% CI: 1.28–8.95) at baseline were significantly associated with unfavourable outcome (Table 4). In subgroup analysis restricted to people with HIV (PWH), a baseline CD4 count <50 cells/mm^3^ was independently associated with increased risk of an unfavourable outcome (aOR, 95% CI: 3.62, 1.24–10.52, Supplementary Table 6).

**Table 4.** Predictors of Unfavorable Outcome (TB-free Survival NOT Achieved) at 18 Months (Binary Logistic Regression Model, Total n = 206)

Explanatory Variables	Crude		Adjusted	
	OR (95% CI)	*P* Value	aOR (95% CI)	*P* Value
HIV (Ref = HIV-negative)				
HIV positive, VL <50 copies per mL	0.87 (.27–2.76)	.82	0.93 (.25–3.4)	.91
HIV positive, VL = 50–1000 copies per mL	1.11 (.34–3.65)	.86	1.00 (.26–3.82)	1.00
HIV positive, VL >1000 copies per mL	2.08 (.92–4.74)	.08	2.27 (.81–6.36)	.12
Age	1.02 (.99–1.04)	.28	1.05 (1.01–1.08)	.02
Body mass index	0.91 (.84–.99)	.02	0.92 (.83–1.01)	.06
Albumin	0.92 (.88–.97)	.001	0.94 (.88–.99)	.03
Functional status (ECOG score) (Ref = Gr. 0–2)				
Gr. 3–4	0.70 (.25–1.95)	.49	0.23 (.07–.77)	.02
Sputum smear grade (Ref = negative)				
1+	0.84 (.31–2.32)	.74	1.02 (.32–3.19)	.98
2+	1.12 (.44–2.85)	.81	1.30 (.45–3.76)	.63
3+	2.45 (1.09–5.53)	.03	3.38 (1.28–8.95)	.01
Previous DS-TB (Ref = no)				
Yes	1.37 (.72–2.6)	.34		
Treatment interruption ≥2 m (Ref = no)				
Yes	1.89 (.86–4.15)	.11	2.25 (.9–5.61)	.08
Initial care setting (Ref = outpatient)				
Inpatient	2.17 (1.08–4.35)	.03		

Abbreviations: CI, confidence interval; DS-TB, drug-susceptible tuberculosis; ECOG, Eastern Cooperative Oncology Group; HIV, human immunodeficiency virus; Ref, reference category; VL, viral load,

## DISCUSSION

In this observational cohort study, 38% of participants were successfully treated with a 9-month oral regimen for RR-TB in a high HIV-burden TB programme. The most important reasons for regimen failure were switching to individualised regimens, loss to follow-up and death during treatment. Switching to an individualised regimen occurred mainly because of delayed detection of preexisting fluoroquinolone resistance and the inability to exclude extensive lung disease before starting treatment due to challenges with access to radiology services. The proportion with TB-free survival was 63% at 18 months after treatment initiation, with unfavorable outcomes driven by mortality (18%) and loss to follow-up (17%) rather than disease recurrence.

A large proportion of PWH had advanced HIV disease and poor virological control at the start of TB treatment. Unfavorable outcomes (22% vs 15% in HIV-negative) and death (21% vs 12% in HIV-negative) were higher among PWH, though the difference was not statistically significant and the study was not powered for this outcome. After adjustment for potential confounders, HIV positivity, irrespective of baseline virologic control, was not associated with increased time to culture conversion or the risk of unfavourable outcome at 18 months. All PWH were initiated (or reinitiated) on ART after starting RR-TB treatment, which potentially explains the similar outcomes to HIV-negative participants. However, deaths appeared to increase among PWH at later time points; reasons for this were not determined in our study but may be a consequence of ART interruption.

High bacterial burden (≥3 smear positivity), older age, and lower albumin were associated with worse microbiological and clinical outcomes. These have been previously identified as risk factors for unfavourable outcomes in other populations with TB [8–10]. In high-burden settings, such markers could serve as a rationale for risk-stratified allocation of scarce resources such as admission to hospital or additional adherence, nutritional and social support. Unexpectedly, worse functional performance (ECOG grade 3–4) at baseline was associated with decreased risk of unfavourable outcome. This is likely a consequence of unmeasured confounders, such as enhanced care provision. For example, almost all these participants (30/33, 91%) underwent an initial period of in-hospital care.

Nearly one-third (73/248, 29%) of participants experienced a prolonged (≥2 consecutive months) treatment interruption at some point during follow-up, although more than half (44/73, 60.3%) returned to care. The high rate of treatment interruption is a major concern. Due to its long half-life, treatment interruptions may lead to periods of effective bedaquiline-monotherapy in partially treated patients, potentially giving rise to resistance, particularly among patients who remain culture-positive after treatment discontinuation [11–13]. This is supported by emerging data from South Africa suggesting a rise in bedaquiline resistance [11, 14, 15]. In our study, 23% (10/44) who returned to care after prolonged treatment interruption were sputum culture positive, one of whom had acquired bedaquiline resistance. Despite the expectation that the shorter duration and lower pill burden of the BPaL/M regimen will lead to a reduction in loss to follow-up, TB programs should have strategies to support engagement in care, trace patients who disengage and enhance laboratory capacity to perform bedaquiline DST.

Some findings from our study differ from SA-NTP data, which informed the inclusion of this regimen in WHO guidelines in 2022 [5]. The SA-NTP reported 64% WHO-defined treatment success at 24 months after treatment initiation, where 20% died, 1% had treatment failure or recurrence, and 15% were lost to follow-up. Bacteriological failure (2%) and late mortality (18% at 18 months) were similar in our study, although treatment success was much lower (38%). The SA-NTP analysis was restricted to patients with a regimen duration ≤12 months, therefore excluding patients who started the oral SCR but switched to longer individualised regimens due to pre-existing contraindications. A comparison to the mITT population from our study (49% success at end of treatment) may therefore be more appropriate but is still worse than the SA-NTP. The worse outcomes observed in our study are driven by the higher number of participants lost to follow-up, potentially more accurately ascertained in a prospective cohort.

Our study has some limitations. It was conducted at a single facility with local factors, such as limited access to radiography services, impacting results and limiting generalizability. Some variables that may affect outcomes could not be measured, including chest radiography and long-term treatment adherence. A limitation of the TB-free survival outcomes framework is that untraceable individuals were considered “unassessable,” reducing power and potentially introducing bias. To mitigate this, a multi-pronged strategy, including a National Death Register search, was used to minimize the number unassessable. Furthermore, “TB-free” status is based on a single (most recent) culture in contrast to the WHO definition of “cure” based on 2 consecutive negative cultures ≥7 days apart without a subsequent positive culture (ie, stable culture conversion). “TB-free” cannot therefore be viewed as equivalent to “cure” but provides a pragmatic provider- and patient-centred outcome that is simpler to ascertain and could be applied in a programmatic setting.

In conclusion, a shorter oral regimen for RR-TB performed poorly in a high HIV-burden TB program, with low rates of successful regimen completion, high mortality and disengagement from care. However, culture conversion was rapidly achieved in most patients who started the regimen. Outcomes did not differ significantly by HIV status. Strategies to support the implementation of effective new regimens, like BPaL/M, are needed to translate outcomes from clinical trials into practice.

## Supplementary Material

ciaf112_Supplementary_Data

## Supplementary Data

Supplementary materials are available at *Clinical Infectious Diseases* online. Consisting of data provided by the authors to benefit the reader, the posted materials are not copyedited and are the sole responsibility of the authors, so questions or comments should be addressed to the corresponding author.

## Notes

***Author Contributions.*** J. A. M. S.: Conception and design of the study, acquisition of data, analysis and interpretation of data, and participation in drafting and revision of the manuscript. J. J. K.: Acquisition of data, analysis and interpretation of data, and participation in drafting and revision of the manuscript. S. F. M.: Analysis and interpretation of data, and participation in drafting and revision of the manuscript. N. N. M.: Acquisition of data. C. C. H.: Acquisition of data. G. G. Ma.: Interpretation of data, and participation in drafting and revision of the manuscript. R. R. W.: Conception and design of the study, interpretation of data, and participation in drafting and revision of the manuscript. G. G. Me.: Conception and design of the study, interpretation of data, and participation in drafting and revision of the manuscript. S. S. W.: Conception and design of the study, acquisition of data, analysis and interpretation of data, and participation in drafting and revision of the manuscript.

***Acknowledgments.*** The authors gratefully acknowledge the assistance by Tracy Glass from the South African Medical Research Councill with national death registry data linkage.

***Data availability.*** Data are not publicly available but can be made available upon request from the authors.

***Financial support.*** This work was supported by the South African Medical Research Council (grant number MRC-RFA-SHIP 02-2018); the Swedish Heart-Lung Foundation (grant number 20220859 to J. J. K.); and the Swedish Society of Medicine (grant number SLS-985976 to J. J. K). S. S. W. was supported by the National Institutes of Health (grant number U01AI170426) and the Bill & Melinda Gates Foundation (INV-052110).

***Potential conflicts of interest**.* The authors: No reported conflicts of interest. All authors have submitted the ICMJE Form for Disclosure of Potential Conflicts of Interest. Conflicts that the editors consider relevant to the content of the manuscript have been disclosed.
