# Correction to: Treatment Outcomes With an Oral Short Course Regimen for Rifampicin-resistant Tuberculosis in a High HIV Prevalence, Programmatic Setting in South Africa

**DOI:** 10.1093/cid/ciaf711

**Published:** 2026-01-09

This is a correction to: Jacob A M Stadler, Johanna Kuhlin, Síle F Molloy, Nomfuneko Mtwa, Cindy Hayes, Gary Maartens, Robin Warren, Graeme Meintjes, Sean Wasserman, Treatment Outcomes With an Oral Short Course Regimen for Rifampicin-resistant Tuberculosis in a High HIV Prevalence, Programmatic Setting in South Africa, *Clinical Infectious Diseases*, Volume 81, Issue 4, 15 October 2025, Pages e153–e162, https://doi.org/10.1093/cid/ciaf112

The originally published version of this manuscript omitted to include author David Stead. This error has been corrected in the article.

