# Supplementary material for: Treatment Outcomes With an Oral Short Course Regimen for Rifampicin-resistant Tuberculosis in a High HIV Prevalence, Programmatic Setting in South Africa
Source: Clin Infect Dis. 2025 May 9;81(4):e153–62. doi: 10.1093/cid/ciaf112 (PMC12596408; doi:10.1093/cid/ciaf112)
Supplement: ciaf112_Supplementary_Data [file ciaf112_supplementary_data.zip › Stadler et al_Supplemental data_27Feb2025.docx]

**Supplemental data online for publication:**

Table S1. Outcome definitions for “TB-free survival”.

| TB-free survival is determined by assessing the following three elements at a specified point in time:  1) vital status (alive/dead)  2) last sputum culture result (Mtb positive/NOT Mtb positive)  3) treatment complete ^(A)^ OR in care ^(B)^  TB-free survival is a binary outcome (Achieved/Not Achieved) with the following sub-categories/components:  TB-free survival achieved:  Alive, TB-free, Treatment complete  Alive, TB-free, In care (i.e. treatment ongoing)  TB-free survival NOT achieved:  NOT TB-free (i.e. last culture Mtb positive), but alive & in care (i.e. treatment ongoing)  Died  Unassessable ^(C)^  Treatment NOT completed & NOT in care (i.e. lost to follow-up) |
| --- |
| ^(A)^ Treatment complete = Recommended duration of treatment completed according to local guidelines OR full duration of treatment not completed, but a decision was taken by a health care provider that no further treatment is required, irrespective of the reason.  ^(B)^ In care = Treatment ongoing OR treatment incomplete but currently halted, irrespective of the reason, with attendance of ongoing follow-up visits (i.e. considering recommencement of treatment).  ^(C)^ One or more of the following apply:  Unknown vital status ^(D)^  Unknown last sputum culture status including if alive, but treatment NOT complete and NOT in care (i.e. recent sputum culture status could not be assessed)  Unknown if treatment complete/in care (e.g. alive, last culture not Mtb positive, unknown if in care/treatment complete).  ^(D)^ Participants with unknown vital status at month 18 (due to being uncontactable and no medical records confirming vital status) following a successful primary outcome (cured or treatment completed) at the end of treatment with the oral SCR and where the National Death Registry search yielded no evidence that they died, were assigned an outcome of “TB-free survival achieved” instead of “Unassessable”. |

Table S2. Composition of initial (standardised) and secondary (individualised) TB regimens, in case of regimen change^A^, through month 18 of follow-up.

| Drug | Initial regimen  N = 248 | Secondary regimen  N = 98 |
| --- | --- | --- |
| Bedaquiline | 245 (98.8%) ^B^ | 85 (86.7%) |
| Clofazimine | 248 (100%) | 96 (98.0%) |
| Linezolid | 217 (87.5%) ^B^ | 76 (77.6%) |
| Levofloxacin | 248 (100%) | 78 (79.6%) |
| Isoniazid | 248 (100%) | 33 (33.7%) |
| Pyrazinamide | 248 (100%) | 21 (21.4%) |
| Ethambutol | 246 (99.2%) | 4 (4.1%) |
| Terizidone | 0 (0%) | 84 (85.7%) |
| Delamanid | 0 (0%) | 38 (38.8%) |
| Para-amino salicylic acid | 0 (0%) | 11 (11.2%) |
| Moxifloxacin | 0 (0%) | 5 (5.1%) |
| Number of drugs, median (min; max) | 7 (5; 7) | 5 (3; 8) |

^A^ Regimen change was defined as permanent discontinuation or change of two or more drugs. Substitution with an equivalent drug (e.g. moxifloxacin for levofloxacin) was not considered a drug change.

^B^ In some patients, linezolid and bedaquiline were initially omitted due to severe anaemia and QT prolongation, respectively, and introduced later if possible.

Table S3. Initial and subsequent antiretroviral treatment (ART) regimens used by people with HIV. (n = 173).

| Initial ART regimen^A^ | | Subsequent ART regimen ^B^ | |
| --- | --- | --- | --- |
| Regimen | n = 173 | Regimen | n = 21 |
| 3TC/TDF/DTG | 124 (71.1%) | 3TC/TDF/DTG | 6 (28.6%) |
| 3TC/ABC/DTG | 3 (1.7%) | 3TC/ABC/DTG | 7 (33.3%) |
| 3TC/AZT/DTG | 1 (0.6%) | 3TC/AZT/DTG | 1 (4.8%) |
| FTC/TDF/EFV | 3 (1.7%) | 3TC/TDF/LPVr | 3 (14.3%) |
| FTC/TDF/LPVr | 15 (8.7%) | FTC/TDF/LPVr | 1 (4.8%) |
| 3TC/TDF/LPVr | 10 (5.8%) | 3TC/ABC/LPVr | 1 (4.8%) |
| 3TC/ABC/LVPr | 9 (5.8%) | FTC/TDF/ATVr | 1 (4.8%) |
| 3TC/AZT/LPVr | 1 (0.6%) | FTC/TDF/EFV | 1 (4.8%) |
| 3TC/TDF/ATVr | 1 (0.6%) |  |  |
| 3TC/ABC/ATVr | 1 (0.6%) |  |  |
| 3TC/ABC/NVP* | 1 (0.6%) |  |  |
| 3TC/TDF/ABC/DTG** | 1 (0.6%) |  |  |
| Missing data | 3 (1.7%) |  |  |

3TC = lamivudine; ABC = abacavir; AZT = zidovudine; ATVr = atazanavir-ritonavir; DTG = dolutegravir; EFV = efavirenz; FTC = Emtricitabine; LPVr = lopinavir-ritonavir; NVP = nevirapine; TDF = tenofovir

*^A^ The regimen taken at the time of TB treatment initiation or, if not on ART at the time (i.e. ART naïve or interrupted), the regimen that was subsequently initiated.*

*^B^ If the initial regimen was changed; if multiple regimen changes, only the first change is reflected.*

* This participant was pregnant.; ** This participant had concomitant Hepatitis B.

Table S4: Treatment outcomes* for the oral SCR at the end of treatment with the regimen, amongst the modified intention-to-treat population (n = 183). *2021 WHO Reporting Framework.

| *Values given as n (%)* | *All*  *N = 183 (100)* | *HIV-negative*  *n = 54 (29.5)* | *HIV- positive*  *n = 129 (70.5)* | *P - value* |
| --- | --- | --- | --- | --- |
| Regimen successful (Cured or Treatment completed) | 90 (49.2) | 31 (57.4) | 62 (48.1) | P = 0.25 |
| Regimen unsuccessful | 93 (50.8) | 23 (42.6) | 67 (51.9) | P = 0.25 |
| Died | 20 (10.9) | 5 (9.3) | 15 (11.6) | P = 0.64 |
| Lost to follow up | 48 (26.2) | 13 (24.1) | 35 (27.1) | P = 0.67 |
| Treatment failed (regimen changed permanently) | 22 (12.0) | 5 (9.3) | 17 (13.2) | P = 0.46 |
| Poor clinical and/or bacteriological response | 6 (3.3) | 1 (1.9) | 5 (3.9) |  |
| Adverse drug reaction | 8 (4.4) | 2 (3.7) | 6 (4.7) |  |
| Acquired resistance to drugs in the regimen | 1 (0.5) | 0 (0.0) | 1 (0.8) |  |
| Other | 7 (3.8) | 2 (3.7) | 5 (3.9) |  |

Table S5: Factors associated with sputum culture conversion rate. (Cox proportional hazards model, total n = 203)

| *Explanatory variables* | *Univariate association* | | *Final multivariate model* | | |
| --- | --- | --- | --- | --- | --- |
|  | *HR (95% CI)* | *P-value* | *aHR (95% CI)* | *P-value* |  |
| **HIV** (Ref = HIV-negative) |  |  |  |  |  |
| HIV positive, VL<50 copies per mL | 1.02 (0.65 - 1.61) | 0.92 | 1.06 (0.68-1.66) | 0.78 |  |
| HIV positive, VL = 50-1000 copies per mL | 1.26 (0.69 - 2.27) | 0.45 | 1.36 (0.78-2.38) | 0.27 |  |
| HIV positive, VL>1000 copies per mL | 0.86 (0.57 - 1.30) | 0.48 | 0.92 (0.62-1.35) | 0.66 |  |
| **Sex** (Ref = Male) |  |  |  |  |  |
| Female | 1.14 (0.83 - 1.58) | 0.41 |  |  |  |
| **Diabetes** (Ref = No) |  |  |  |  |  |
| Yes | 0.73 (0.43 - 1.22) | 0.22 | 0.75 (0.45-1.25) | 0.26 |  |
| **FQ sensitivity** (Ref = Susceptible) |  |  |  |  |  |
| Resistant | 0.65 (0.4-1.05) | 0.08 | 0.65 (0.4-1.05) | 0.08 |  |
| **Sputum smear grade** (Ref=Negative) |  |  |  |  |  |
| 1+ | 0.89 (0.55 - 1.43) | 0.62 | 0.83 (0.53-1.28) | 0.40 |  |
| 2+ | 0.70 (0.44 - 1.12) | 0.14 | 0.66 (0.43-1.01) | 0.05 |  |
| 3+ | 0.69 (0.43 - 1.09) | 0.11 | 0.64 (0.42-0.95) | 0.03 |  |
| **GeneXpert cycle threshold** | 1.03 (0.94 - 1.12) | 0.54 |  |  |  |
| **Previous DS-TB** (Ref = No) |  |  |  |  |  |
| Yes | 1.12 (0.82 - 1.52) | 0.48 |  |  |  |
| **Initial care setting** (Ref = Outpatient) |  |  |  |  |  |
| Inpatient | 1.49 (1.06 - 2.11) | 0.02 | 1.53 (1.09-2.15) | 0.01 |  |

Ref = reference category; VL = viral load; FQ = fluoroquinolone

Table S6. Predictors of unfavourable outcome (TB-free survival NOT achieved) at 18 months among people with HIV. (Binary logistic regression model, total n=147)

| *Explanatory variables* | *Crude* | | *Adjusted* | | |
| --- | --- | --- | --- | --- | --- |
|  | *OR (95% CI)* | *P-value* | *aOR (95% CI)* | *P-value* |  |
| CD4 cell count (Ref: > 200 cells/mm^3^) |  |  |  |  |  |
| < 50 cells/mm^3^ | 3.62 (1.24-10.52) | 0.02 | 3.62 (1.24-10.52) | 0.02 |  |
| 50 – 200 cells/mm^3^ | 2.37 (0.79-7.15) | 0.12 | 2.37 (0.79-7.15) | 0.12 |  |
| HIV viral load (Ref: < 50 copies per mL) |  |  |  |  |  |
| 50-1000 copies per mL | 1.28 (0.32-5.14) | 0.73 |  |  |  |
| >1000 copies per mL | 2.39 (0.78-7.31) | 0.13 |  |  |  |
| Timing of ART initiation (Ref = Already on ART) |  |  |  |  |  |
| ≤ 4 weeks after TB treatment | 2.12 (0.96-4.68) | 0.06 |  |  |  |
| > 4 weeks after TB treatment | 1.65 (0.39-6.98) | 0.50 |  |  |  |
| Age, years | 1.00 (0.97-1.04) | 0.81 |  |  |  |
| Body mass index | 0.91 (0.830-1.00) | 0.05 |  |  |  |
| Care setting (Ref = Outpatient) |  |  |  |  |  |
| Inpatient | 2.53 (1.06-6.05) | 0.04 |  |  |  |

**Supplemental figure legend**

Figure S1. Baseline drug resistance patterns from programmatic drug susceptibility testing data.

Alt text: A pie graph showing proportions of resistance patterns among *M tuberculosis* isolates collected at baseline, with rifampicin-resistance detected on GeneXpert alone in 31.9%; MDR-TB in 36.7%; and fluoroquinolone resistance in 8.9%.

**Footer for figure S1**

RR (GXP only) = Rifampicin-resistant based on GeneXpert Mtb/Rif only (i.e. no further drug suscetibility tests were performed); RIF-mono = rifampicin-monoresistance (i.e. isoniazid susceptible); MDR = multidrug-resistant (i.e. resistant to rifampicin and isoniazid with either *katG* or *inhA* mutations); MDR+inhDM = multidrug-resistant with dual (*katG* and *inhA)* mutations; MDR+SLI = multidrug-resistant with additional resistance to second-line injectables; MDR+FQ = multidrug-resistant with additional resistance to fluoroquinolones; MDR+SLI+FQ = multidrug-resistant with additional resistance to second-line injectables and fluoroquinolones; MDR+SLI+FQ+BDQ = multidrug-resistant with additional resistance to second-line injectables, fluoroquinolones and bedaquiline.
