# Supplementary figures and images for: Treatment Outcomes With an Oral Short Course Regimen for Rifampicin-resistant Tuberculosis in a High HIV Prevalence, Programmatic Setting in South Africa
Source: Clin Infect Dis. 2025 May 9;81(4):e153–62. doi: 10.1093/cid/ciaf112 (PMC12596408; doi:10.1093/cid/ciaf112)

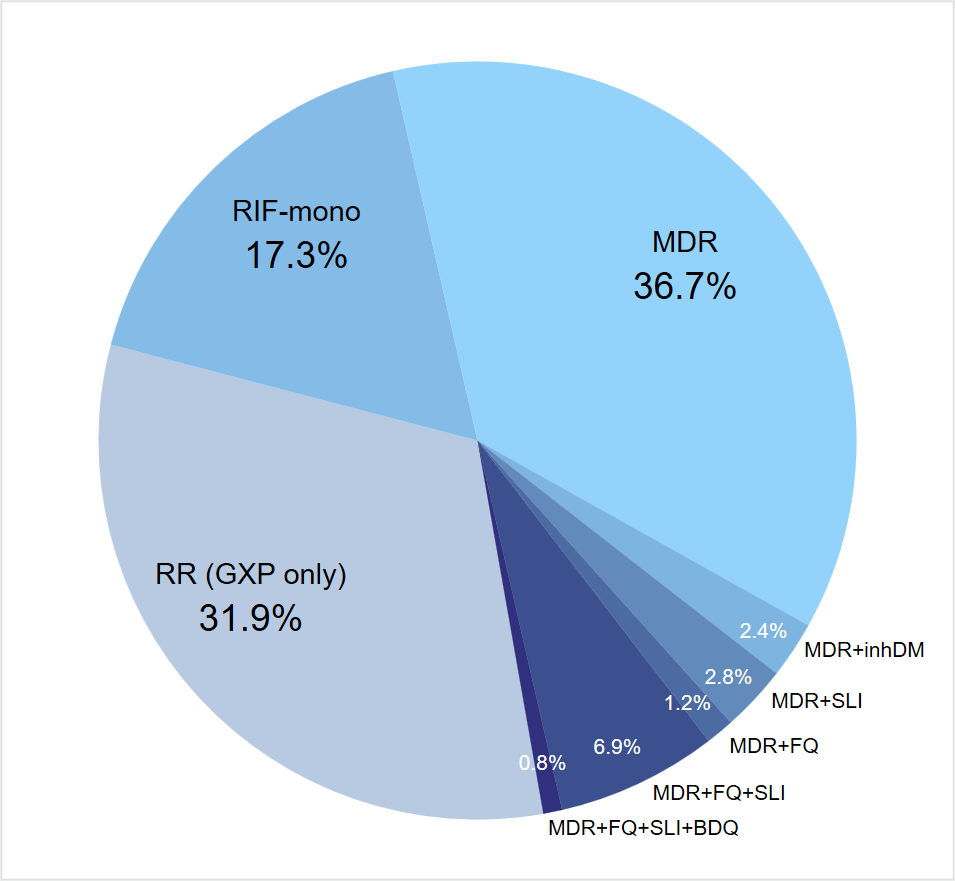

Supplement: ciaf112_Supplementary_Data [file ciaf112_supplementary_data.zip › Figure S1.tif]
